# Clinical characteristics and risk factors for in‐hospital mortality of lung cancer patients with COVID‐19: A multicenter, retrospective, cohort study

**DOI:** 10.1111/1759-7714.13710

**Published:** 2020-11-03

**Authors:** Lei Nie, Kai Dai, Jiang Wu, Xia Zhou, Junjun Hu, Chao Zhang, Yan Zhan, Yu Song, Wen Fan, Zhimin Hu, Hongshan Yang, Qiong Yang, Dongde Wu, Fajiu Li, Daoyuan Li, Rui Nie

**Affiliations:** ^1^ Department of Hepatobiliary and Pancreatic Surgery Hubei Cancer Hospital, Tongji Medical College, Huazhong University of Science and Technology Wuhan China; ^2^ Department of Infectious Diseases Renmin Hospital of Wuhan University Wuhan China; ^3^ Department of Hematology Zhongnan Hospital of Wuhan University Wuhan China; ^4^ Department of Respiratory and Critical Care Medicine Wuhan Jinyintan Hospital Wuhan China; ^5^ Department of Surgery Wuhan NO. 7 Hospital Wuhan China; ^6^ Department of Hepatobiliary Surgery Wuhan NO.1 hospital Wuhan China; ^7^ Department of rehabilitation medicine Xiangyang Central Hospital, Affiliated Hospital of Hubei University of Arts and Science Xiangyang China; ^8^ Department of Respiratory and Critical Care Medicine The Central Hospital of Wuhan Wuhan China; ^9^ Department of General Medicine The First People's Hospital of Jingzhou Affiliated No. 1 Hospital of Changjiang University Jingzhou China; ^10^ Endoscopy Center Wuhan Pulmonary Hospital, Wuhan Institute for Tuberculosis Control Wuhan China; ^11^ Department of Medical Oncology Affiliated Xiaogan Hospital of Wuhan University of Science and Technology Xiaogan China; ^12^ Department of Obstetrics Hubei Maternal and Child Health Hospital Wuhan China; ^13^ Department of Pulmonary and Critical Care Medicine Affiliated Hospital of Jianghan University Wuhan China; ^14^ Department of Art and Design Hubei University of Technology Engineering and Technology College Wuhan China; ^15^ Reproductive Medicine Center Tongji Hospital, Tongji Medical College, Huazhong University of Science and Technology Wuhan China

**Keywords:** Clinical features, COVID‐19, lung cancer, mortality, risk factors

## Abstract

**Background:**

Data on clinical, laboratory, and radiographic characteristics and risk factors for in‐hospital mortality of lung cancer patients with COVID‐19 are scarce. Here, we aimed to characterize the early clinical features of lung cancer patients with COVID‐19 and identify risk factors associated with in‐hospital mortality.

**Methods:**

All consecutive lung cancer patients with laboratory‐confirmed COVID‐19 admitted to 12 hospitals in Hubei province, China, from 3 January to 6 May 2020 were included in the study. Patients without definite clinical outcomes during the period were excluded. Data on initial clinical, laboratory and radiographic findings were compared between survivors and nonsurvivors. Univariable and multivariable logistic regression analyses were used to explore the risk factors associated with in‐hospital mortality.

**Results:**

Of the 45 lung cancer patients (median [interquartile range] age, 66 [58–74] years; 68.9% males) included, 34 (75.6%) discharged and 11 (24.4%) died. Fever (73.3%) and cough (53.3%) were the dominant initial symptoms, and respiratory symptoms were common. Lung cancer patients also presented atypical appearances of COVID‐19. In the multivariable analysis, prolonged prolongation prothrombin time (PT) (OR = 2.1, 95% CI: 1.00–4.41, *P* = 0.0497) and elevated high sensitivity cardiac troponin I (hs‐TNI) (OR = 7.65, 95% CI: 1.24–47.39, *P* = 0.0287) were associated with an increased risk of in‐hospital mortality.

**Conclusions:**

Lung cancer patients with COVID‐19 have high in‐hospital mortality. Prolonged PT and elevated hs‐TNI are independent risk factors for in‐hospital mortality of lung cancer patients with COVID‐19.

**Key points:**

**Significant findings of the study:**

- Lung cancer patients with COVID‐19 have atypical early symptoms and imaging features.
- The prolonged prothrombin time and elevated high sensitivity cardiac troponin I are independent risk factors for in‐hospital mortality of lung cancer patients with COVID‐19.

**What this study adds:**

This study characterizes the early clinical features of lung cancer patients with COVID‐19 in China, and identifies the risk factors associated with in‐hospital mortality of lung cancer patients with COVID‐19.

## Graphical abstract

Lung cancer patients with COVID‐19 have high in‐hospital mortality with unique early symptoms and imaging features. Prolonged PT and elevated hs‐TNI are independent risk factors for in‐hospital mortality of lung cancer patients with COVID‐19. They may be used to help clinicians predict prognosis at an early stage for lung cancer patients with COVID‐19.

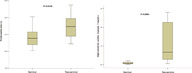

## Introduction

Coronavirus disease 2019 (COVID‐19) caused by severe acute respiratory syndrome coronavirus 2 (SARS‐CoV‐2) is now a global health, information and increasingly a wider socioeconomic crisis.^1^ Patients with cancer have been shown to have a high risk of morbidity and mortality from COVID‐19.^2^, ^3^ Lung cancer represents a unique scenario of cumulative risk factors for COVID‐19 morbidity and mortality, including older age, multiple comorbidities, long smoking history, tumor‐related lung damage, as well as the unavoidable addition of treatment‐related immunosuppression.^4^, ^5^, ^6^ Moreover, both mechanical tumor obstruction and previous lung resection surgery can cause defective pulmonary architecture, which may make lung cancer patients more predisposed to SARS‐CoV‐2 infection with a poor prognosis if COVID‐19 is suspected. However, data specifically focused on mortality, and the underlying risk factors for lung cancer patients with COVID‐19 are scarce. Rogado *et al*. first reported 17 lung cancer patients with COVID‐19 in Spain, when nine (52.9%) patients died.^6^ However, the laboratory and radiographic findings of these patients were not described in the study.^6^ Garassino *et al*. reported that only smoking history was associated with increased risk of death of patients with thoracic malignancies and COVID‐19.^5^ However, this was a registry‐based study and clinical data, such as laboratory and radiographic characteristics of the patients, were not specified.^5^

In this cohort study, we aimed to characterize the early clinical features of lung cancer patients with COVID‐19 in China, and identify the risk factors associated with in‐hospital mortality of lung cancer patients with COVID‐19.

## Methods

### Study design and participants

This retrospective study was conducted at 12 hospitals in Hubei province, China. All consecutive hospitalized patients diagnosed with lung cancer at any time and COVID‐19 on admission, or during hospitalization from 3 January to 6 May 2020, were identified. Patients with laboratory‐confirmed COVID‐19 and a definite clinical outcome (discharge or death) were included in the analysis. Laboratory‐confirmed COVID‐19 was defined when SARS‐CoV‐2 nucleic acid was positive as detected by reverse transcription‐polymerase chain reaction (RT‐PCR) assay, as described previously.^7^ Patients who were transferred to other hospitals were excluded. The Research Ethics Commissions of Jinyintan Hospital approved this study and granted a waiver of informed consent from study participants (No.KY‐2020‐73.01).

### Procedures of data collection and definitions

A trained team of physicians collected data from the electronic medical records using a standardized data collection form. Data included age, sex, smoking status, comorbidities, laboratory, radiographic, and electrocardiographic findings, pathology of lung cancer, active anticancer therapy (within four weeks of COVID‐19 diagnosis), cancer status (present vs. in remission), onset date, clinical manifestations and treatment of COVID‐19 and outcomes. Current lung cancer patients included all cases with newly diagnosed, partial remission, progressive, or recurrent cancer, while lung cancer patients in remission included those treated who went into complete remission.^8^ The date of COVID‐19 onset was defined as the day when COVID‐19‐related symptoms such as fever, cough, chest tightness, shortness of breath, etc. were first noticed, and the severity of the disease was classified according to WHO guidelines.^9^ Sepsis was defined as the Third International Consensus Definitions for Sepsis.^10^ The diagnosis of acute respiratory distress syndrome (ARDS) was based on the Berlin Definition.^11^ Patients were considered to have an acute cardiac injury if serum levels of high sensitivity cardiac troponin I (hs‐TNI) were above the 99th percentile upper reference limit, or if new abnormalities were revealed on electrocardiography (ECG) and echocardiography.^12^

Chest computed tomography (CT) images were independently assessed for the presence and distribution of abnormalities by three chest radiologists. Ground‐glass opacity (GGO) was defined as hazy increased opacity in the lung, with the preservation of bronchial and vascular margins.^13^, ^14^ When there was a discrepancy, a final decision was reached by consensus.

### Outcomes

Outcomes included in‐hospital mortality and discharge. The criteria for discharge were normal body temperature for at least three days, clinical remission of respiratory symptoms, substantial resolution of inflammation as shown by chest radiography, and two consecutive negative results for SARS‐CoV‐2 RNA obtained at least 24 hours apart.^7^

### Statistical analysis

Continuous variables are presented as median and interquartile ranges (IQR), and categorical variables are expressed as counts and percentages. Continuous variables to compare the differences between survivors and nonsurvivors were assessed using a Wilcoxon sum test, and categorical variables were analyzed using Chi‐square analysis or Fisher's exact test as appropriate; odds ratio (OR) and 95% confidence interval (CI) were calculated.

To explore the risk factors for in‐hospital mortality, univariate and multivariate logistic regression models were used. Multivariate logistic regression was performed, first by adjusting for the factors with a *P‐*value of <0.2 in univariate analysis, and then limiting final multivariate models to risk factors or confounders that were statistically significant in analyses. No imputation was made for missing data. If the RT‐PCR assay for SARS‐CoV‐2 was positive when the patient died, the date of death was used as the end date. All statistical tests were two‐sided, and a *P*‐value of <0.05 was considered statistically significant. For all analyses, SAS 9.3 (SAS Institute, Cary, NC) was used.

## Results

### Demographic, clinical, and tumor characteristics

From 3 January 2020, to 6 May 2020, 50 patients with lung cancer were identified from 11 660 patients clinically diagnosed with COVID‐19 and admitted to 12 hospitals in Hubei Province. Due to the newly constructed hospitals during the epidemic of COVID‐19, some patients, including lung cancer patients with COVID‐19, were transferred to the new hospitals for a reasonable allocation of medical resources. Five patients were then excluded, and thus 45 patients were included in the analysis. As of 6 May 2020, 34 patients were discharged, and 11 died, with an in‐hospital mortality rate of 24.4%.

Demographic, clinical, and tumor characteristics for patients are shown in Table 1. The median (IQR) age of the patients was 66 (58–74) years. There was a significant difference in age between survivors and nonsurvivors (61.5 [57.0–72.0] vs. 70.0 [67.0–80.0], *P* = 0.0207), but no difference in the sex ratio (*P* = 0.1318). Comorbidities were present in 30 (66.7%) patients, with hypertension being the most common comorbidity, followed by coronary artery disease and diabetes. The prevalence of hypertension was higher in nonsurvivors than in survivors (63.6% vs. 23.5%, *P* = 0.0256).

**Table 1:** Patient demographic, clinical, and tumor characteristics

	Total *N* = 45	Survivor *N* = 34	Nonsurvivor *N* = 11	*P‑*value
Age, years	66.0 (58.0–74.0)	61.5 (57.0–72.0)	70.0 (67.0–80.0)	0.0207
Sex				0.1318
Male	31 (68.9)	21 (61.8)	10 (90.9)	
Female	14 (31.1)	13 (38.2)	1 (9.1)	
History of smoking	11 (24.4)	8 (23.5)	3 (27.3)	1.0000
Comorbidity				
Hypertension	15 (33.3)	8 (23.5)	7 (63.6)	0.0256
Coronary artery disease	7 (15.6)	4 (11.8)	3 (27.3)	0.3368
Diabetes	6 (13.3)	3 (8.8)	3 (27.3)	0.1459
Other	19 (42.2)	13 (38.2)	6 (54.5)	0.4851
Early symptoms of COVID‐19				
Fever	33 (73.3)	25 (73.5)	8 (72.7)	1.0000
Cough	24 (53.3)	18 (52.9)	6 (54.5)	1.0000
Chest tightness	8 (17.8)	6 (17.6)	2 (18.2)	1.0000
Shortness of breath	7 (15.6)	6 (17.6)	1 (9.1)	0.6628
Fatigue	6 (13.3)	5 (14.7)	1 (9.1)	1.0000
Sputum production	5 (11.1)	2 (5.9)	3 (27.3)	0.0853
COVID‐19 disease severity				<0.0001
Mild	4 (8.9)	4 (11.8)	0 (0.0)	
Moderate	18 (40.0)	18 (52.9)	0 (0.0)	
Severe	12 (26.7)	12 (35.3)	0 (0.0)	
Critical	11 (24.4)	0 (0.0)	11 (100.0)	
Time from COVID‐19 onset to hospital admission, days	10.0 (6.5–14.5)	10.0 (7.0–18.0)	8.0 (2.0–12.0)	0.0724
Type of lung cancer				0.0468
Lung adenocarcinoma	10 (22.2)	6 (17.6)	4 (36.4)	
Lung squamous cell cancer	5 (11.1)	4 (11.8)	1 (9.1)	
Small cell lung cancer	2 (4.4)	0 (0)	2 (18.2)	
No information	28 (62.2)	24 (70.6)	4 (36.4)	
Prior thoracic surgery	19(42.2)	15(44.1)	4(36.4)	0.7363
Cancer status				0.4578
Remission	14 (31.1)	12 (35.3)	2 (18.2)	
Present	31 (68.9)	22 (64.7)	9 (81.8)	
Time from lung cancer to COVID‐19, years^†^	1.0 (0.0–3.0)	1.0 (0.0–3.0)	1.0 (1.0–1.0)	0.9126
Cancer treatment within four weeks of COVID‐19 diagnosis				
Lung resection surgery	3 (6.7)	2 (5.9)	1 (9.1)	1.000
Chemotherapy	4 (8.9)	2 (5.9)	2 (18.2)	0.2470
Treatment with immune checkpoint inhibitors	4 (8.9)	3 (8.8)	1 (9.1)	1.0000

Data are presented as the median (interquartile), or number (%), where appropriate.

^†^ Times from the diagnosis of lung cancer to the diagnosis of COVID‐19.

COVID‐19, coronavirus disease 2019.

The two most common early symptoms of COVID‐19 were fever (33 [73.3%]), and cough (24 [53.3%]). Other early symptoms of COVID‐19 included chest tightness (8 [17.8%]), shortness of breath (7 [15.6%]), fatigue (6 [13.3%]) and sputum production (5 [11.1%]), dyspnea (2 [4.4%]), loss of appetite (2 [4.4%]), myalgia (1 [2.2%]), nausea (1 [2.2%]), diarrhea (1 [2.2%]), seizure (1 [2.2%]), dysphagia (1 [2.2%]), headache (1 [2.2%]), and dizziness (1 [2.2%]). Among them, a 68‐year‐old man with a history of epilepsy and small cell lung cancer had a seizure on admission. He presented with respiratory symptoms (sputum production and dyspnea) during hospitalization and died 10 days following admission. There were no significant differences in the early symptoms of COVID‐19 between nonsurvivors and survivors (Table 1).

The most common lung cancer was lung adenocarcinoma (10 [22.2%]), followed by lung squamous cell cancer (5 [11.1%]) and small cell lung cancer (2 [4.4%]). The lung cancer types of 28 patients were not available in the electronic medical records. There were 31 (68.9%) current lung cancer patients and 14 (31.1%) patients in remission. There was no difference between the survivors and nonsurvivors in terms of cancer status and anticancer therapy (Table 1).

### Laboratory, radiographic and electrocardiographic findings

Table 2 shows the laboratory, radiographic and ECG findings on admission. On admission, 33 (73.3%) patients had lymphocytopenia. In univariate analysis, there were significant differences in the lymphocyte count, platelet count, total bilirubin, urea, prothrombin time (PT), D‐dimer, hs‐TNI, and myoglobin between survivors and nonsurvivors.

**Table 2:** Laboratory, radiographic and electrocardiographic findings of patients on admission

	Total *N* = 45	Survivor *N* = 34	Non‐survivor *N* = 11	*P* value
**Laboratory findings**				
White blood cell count ×10^9^/L	5.7 (4.0–7.5)	5.2 (3.9–7.5)	5.9 (4.4–8.4)	0.4840
Neutrophil count ×10^9^/L	3.8 (2.8–6.8)	3.7 (2.8–6.0)	4.4 (2.1–7.4)	0.4129
Lymphocyte count ×10^9^/L	0.8 (0.5–1.2)	0.9 (0.6–1.4)	0.6 (0.2–0.9)	0.0223
Monocyte count ×10^9^/L	0.5 (0.3–0.6)	0.5 (0.4–0.6)	0.3 (0.1–0.6)	0.1424
Red blood cell count ×10^12^/L	4.0 (3.4–4.3)	4.1 (3.7–4.4)	3.5 (3.1–4.1)	0.0725
Hemoglobin, g/L	120.0 (103.0–131.1)	120.5 (106.0–133.0)	120.0 (102.0–131.0)	0.5347
Platelet count ×10^9^/L	187.0 (133.0–245.0)	203.0 (160.0–258.0)	119.0 (66.0–199.0)	0.0028
<125	11 (24.4)	5 (14.7)	6 (54.5)	0.0143
≥125	34 (75.6)	29 (85.3)	5 (45.5)	
ALT, U/L				0.7049
≤40	34 (75.6)	25 (73.5)	9 (81.8)	
>40	11 (24.4)	9 (26.5)	2 (18.2)	
AST, U/L				0.0707
≤40	31 (68.9)	26 (76.5)	5 (45.5)	
>40	14 (31.1)	8 (23.5)	6 (54.5)	
LDH, U/L				
≤245	21 (53.8)	18 (58.1)	3 (37.5)	0.4324
>245	18 (46.2)	13 (41.9)	5 (62.5)	
Total bilirubin, μmol/L				0.0125
≤21	41 (93.2)	33 (100)	8 (72.7)	
>21	3 (6.8)	0 (0)	3 (27.3)	
Urea, mmol/L	5.5 (4.0–8.0)	4.8 (3.7–6.8)	8 (7.2–10.9)	0.0013
Creatinine, μmol/L				0.1434
≤133	42 (93.3)	33 (97.1)	9 (81.8)	
>133	3 (6.7)	1 (2.9)	2 (18.2)	
PT, s	11.8 (10.9–12.9)	11.6 (10.8–12.3)	13.0 (11.8–13.9)	0.0168
APTT, s	29.4 (24.8–32.8)	29.4 (25.0–32.2)	30.0 (23.8–38.1)	0.7311
D‐dimer, mg/L	2.0 (0.7–10.7)	1.5 (0.7–3.8)	19.1 (1.8–37.3)	0.0096
C‐reactive protein, mg/L				0.4101
<10	9 (22)	8 (25.8)	1 (10)	
≥10	32 (78)	23 (74.2)	9 (90)	
Procalcitonin, ng/mL				0.0635
<0.5	35 (83.3)	28 (90.3)	7 (63.6)	
≥0.5	7 (16.7)	3 (9.7)	4 (36.4)	
hs‐TNI				0.0084
Normal	23 (62.2)	20 (76.9)	3 (27.3)	
Elevated	14 (37.8)	6 (23.1)	8 (72.7)	
CK–MB				0.6582
Normal	33 (80.5)	25 (83.3)	8 (72.7)	
Elevated	8 (19.5)	5 (16.7)	3 (27.3)	
Myoglobin, ng/mL	76.9 (35.2–144.6)	52.8 (30.0–87.7)	153.2 (100.1–218.3)	0.0020
NT‐proBNP, pg/mL	254.6 (56.4–1061)	197.4 (37.1–648.8)	1061 (224.0–1383.0)	0.0661
Radiological findings				
GGO with reticular and/or interlobular septal thickening in unilateral lung	4 (8.9)	3 (8.8)	1 (9.1)	1.0000
GGO with reticular and/or interlobular septal thickening in bilateral lungs	40 (88.9)	30 (88.2)	10 (90.9)	1.0000
Pleural effusion	4 (8.9)	2 (5.9)	2 (18.2)	0.2470
Nodule	10 (22.2)	9 (26.5)	1 (9.1)	0.4087
Thoracic lymphadenopathy	5 (11.1)	5 (14.7)	0 (0.0)	0.3131
Pleural thickening	2 (4.4)	2 (5.9)	0 (0.0)	1.0000
Pericardial thickening	3 (6.7)	3 (8.8)	0 (0.0)	0.5651
Electrocardiographic findings	14 (31.1)	8 (23.5)	6 (54.6)	0.0707
Arrhythmia	6 (13.3)	3 (8.8)	3 (27.3)	0.1459
ST segment abnormalities	6 (13.3)	3 (8.8)	3 (27.3)	0.1459
Right bundle branch block	3 (6.7)	2 (5.9)	1 (9.1)	1.0000
QT prolongation	2 (4.4)	1 (2.9)	1 (9.1)	0.4333

Data are presented as the median (interquartile), or number (%), where appropriate.

ALT, alanine aminotransferase; AST, Aspartate aminotransferase; LD, lactate dehydrogenase; PT, prothrombin time; APTT, activated partial thromboplastin time; hs‐TNI, high‐sensitivity troponin I; CK‐MB, creatinine kinase–myocardial band; NT‐proBNP, N‐terminal pro–brain natriuretic peptide; GGO, ground‐glass opacity.

The most common pattern on initial chest CT was GGO with reticular and/or interlobular septal thickening in bilateral lungs (40[88.9%]). Other findings included pleural effusion, nodule, thoracic lymphadenopathy, pleural thickening, and pericardial thickening (Table 2). The median duration from COVID‐19 onset to CT scan was 7.8 days. Radiographic abnormalities did not differ between survivors and nonsurvivors (Table 2).

A total of 14 (31.1%) patients presented with ECG abnormalities on admission. There was no significant difference in ECG abnormalities between survivors and nonsurvivors (Table 2).

### Treatment of COVID‐19 patients and outcomes

Treatment of COVID‐19 patients and outcomes are shown in Table 3. Oxygen therapy use differed significantly between nonsurvivors and survivors (*P* < 0.0001); More patients needed noninvasive and invasive mechanical ventilation in nonsurvivors than survivors (45.5% vs. 8.8%, and 27.3% vs. 0%, respectively). More patients were admitted to the intensive care unit (ICU) in nonsurvivors than survivors (45.5% vs. 2.9%, OR = 27.5, 95% CI: 2.71–278.87).

**Table 3:** Treatments of COVID‐19 and outcomes

	Total *N* = 45	Survivor *N* = 34	Nonsurvivor *N* = 11	*P*‐value
Treatments of COVID‐19				
Oxygen therapy				<0.0001
No oxygen therapy	13 (28.9)	13 (38.2)	0 (0)	
High‐flow nasal cannula oxygen therapy	21 (46.7)	18 (52.9)	3 (27.3)	
Noninvasive mechanical ventilation	8 (17.8)	3 (8.8)	5 (45.5)	
Invasive mechanical ventilation	3 (6.7)	0 (0)	3 (27.3)	
Antibiotics	36 (80)	26 (76.5)	10 (90.9)	0.4157
Antiviral treatment	40 (88.9)	30 (88.2)	10 (90.9)	1.0000
Systematic corticosteroids	12 (26.7)	7 (20.6)	5 (45.5)	0.1306
Intravenous immunoglobulin	5 (11.1)	4 (11.8)	1 (9.1)	1.0000
Renal replacement therapy	1 (2.2)	0 (0)	1 (9.1)	0.2444
Outcomes				
Respiratory failure	12 (26.7)	2 (5.9)	10 (90.9)	<0.0001
Acute cardiac injury	10 (22.2)	4 (11.8)	6 (54.5)	0.0074
ARDS	9 (20.0)	1 (2.9)	8 (72.7)	<0.0001
Heart failure	6 (13.3)	2 (5.9)	4 (36.4)	0.0247
Acute kidney injury	4 (8.9)	0 (0)	4 (36.4)	0.0022
Sepsis	4 (8.9)	0 (0)	4 (36.4)	0.0022
Secondary infection	11 (24.4)	6 (17.6)	5 (45.5)	0.1037
ICU admission	6 (13.3)	1 (2.9)	5 (45.5)	0.0020
Time from illness onset to fever, days	1.0 (1.0–1.0)	1.0 (1.0–1.0)	1.0 (1.0–1.0)	0.4890
Time from illness onset to sputum production, days	1.0 (1.0–2.5)	1.0 (1.0–3.0)	1.0 (1.0–1.5)	0.4175
Time from illness onset to cough, days	1.0 (1.0–2.0)	1.0 (1.0–3.0)	1.0 (1.0–1.0)	0.1066
Time from illness onset to respiratory failure, days	11.0 (8.0–14.0)	9.0 (5.0–13.0)	11.0 (10.0–14.0)	0.4765
Time from illness onset to acute cardiac injury, days	12.0 (7.0–15.0)	12.0 (7.5–20.0)	12.0 (7.0–14.0)	0.5212
Time from illness onset to ARDS, days	12.0 (4.5–14.0)	13.0 (13.0–13.0)	11.0 (1.0–15.0)	0.8252
Time from illness onset to heart failure, days	14.5 (12.0–18.0)	14.0 (12.0–16.0)	15.5 (8.0–32.0)	0.8170
Time from illness onset to ICU admission, days	14.0 (2.0–15.0)	13.0 (13.0–13.0)	14.5 (7.5–16.5)	0.7237
Time from illness onset to corticosteroids treatment, days	12.0 (9.0–23.0)	20.0 (9.0–32.0)	9.5 (8.0–15.0)	0.1066
Duration of treatment with corticosteroids, days	7.0 (6.0–10.0)	6.0 (6.0–10.0)	8.5 (6.0–11.0)	0.7022
Duration of viral shedding after COVID‐19 onset, days	12.0 (7.0–17.5)	11.0 (7.0–17.0)	14.5 (10.0–18.0)	0.4406
Time from illness onset to death or discharge, days	25.5 (19–36.5)	30.5 (22.0–38.0)	18.0 (16.0–22.0)	0.0367

Data are presented as the median (interquartile), or number (%), where appropriate.

ECMO, extracorporeal membrane oxygenation; ICU, intensive care unit; COVID‐19, coronavirus disease 2019; ARDS, acute respiratory distress syndrome; MODS, multiple organ dysfunction syndrome.

Respiratory failure was the most frequently observed complication (26.7%), followed by acute cardiac injury (22.2%), secondary infection (24.4%), ARDS (20.0%), heart failure (13.3%), acute kidney injury (8.9%), and sepsis (8.9%). The incidence of all comorbidities in nonsurvivors was significantly higher than survivors, except for secondary infection (Table 3).

### Risk factors associated with in‐hospital mortality

**Figure 1:**
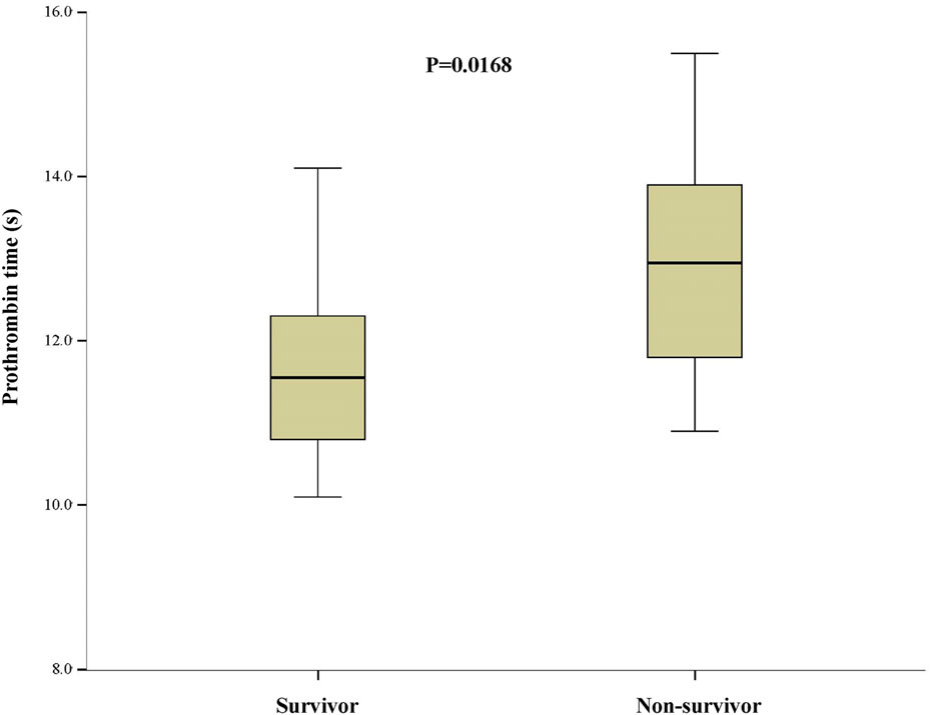
Prothrombin time on admission in lung cancer patients with COVID‐19.

**Figure 2:**
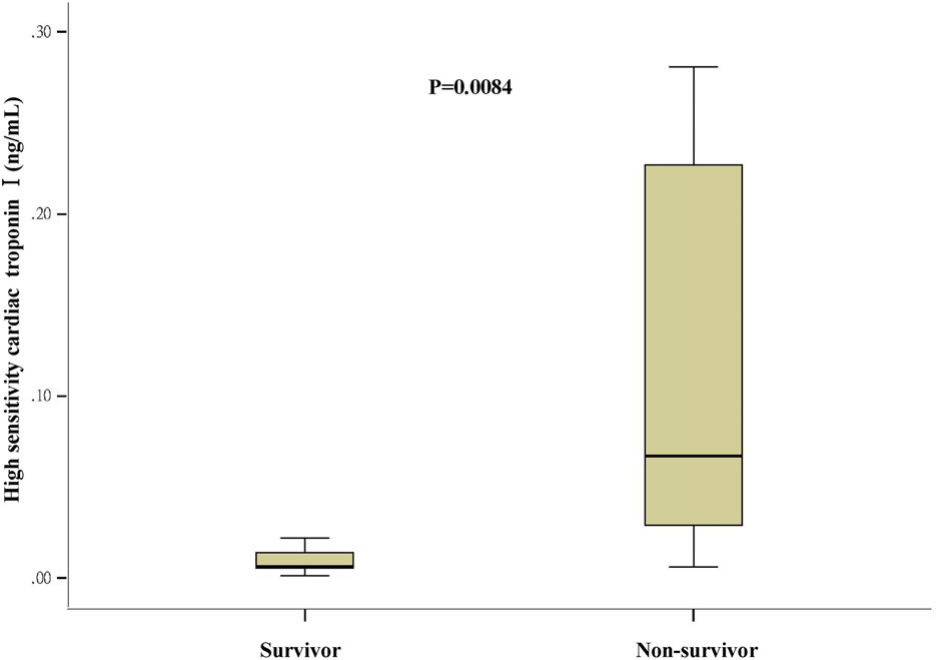
High sensitivity cardiac troponin I on admission in lung cancer patients with COVID‐19.

Univariable analyses revealed that patients who were older (OR = 1.07, 95% CI: 1.01–1.14, *P* = 0.0342), with hypertension (OR = 5.69, 95% CI: 1.32–24.54, *P* = 0.0198), prolonged prothrombin time (PT) on admission (OR = 2.1, 95% CI: 1.14–3.88, *P* = 0.0176), and elevated high sensitivity cardiac troponin I (hs‐TNI) on admission (OR = 8.89, 95% CI, 1.78–44.47) were associated with an increased risk of in‐hospital mortality, whereas elevated platelet count (≥125 × 10^9^/L) (OR = 0.14, 95% CI: 0.03–0.66, *P* = 0.0123) was associated with a decreased risk. In the multivariable analysis, only prolonged PT (OR = 2.1, 95% CI: 1.00–4.41, *P* = 0.0496) (Fig 1) and elevated hs‐TNI (OR = 7.65, 95% CI: 1.24–47.39, *P* = 0.0287) (Fig 2) on admission were associated with an increased risk of in‐hospital mortality.

## Discussion

Our study suggests that the early clinical manifestations, laboratory, and radiographic findings of COVID‐19 in lung cancer patients were similar to those previously reported in patients without cancer in Hubei, especially Wuhan city, China.^12^, ^15^, ^16^ However, lung cancer patients also presented with atypical appearances of COVID‐19. The prolonged PT and elevated hs‐TNI on admission were identified as independent risk factors for the in‐hospital mortality of lung cancer patients with COVID‐19.

Our results show that the in‐hospital mortality rate in lung cancer patients with COVID‐19 in China was 24.4%, while previous studies reported that the fatality rate of noncancer patients in the same age groups with confirmed COVID‐19 in China ranged from 1.3% to 8.0%.^17^, ^18^ Our data indicate a significantly high risk of in‐hospital mortality posed to lung cancer patients with COVID‐19.

The lung cancer symptoms may overlap with symptoms of COVID‐19, since they both directly impair lung function. In our lung cancer cohort, fever and cough were the dominant initial symptoms, and respiratory symptoms were common. Lung cancer patients with COVID‐19 also presented with some nonspecific symptoms, which have been reported in older people or immunosuppressed patients.^9^, ^19^ Also, we observed a 68‐year‐old man with a history of epilepsy and small cell lung cancer who presented with seizures before the onset of respiratory symptoms. The seizures may have occurred due to occult brain metastasis or leptomeningeal carcinomatosis caused by the progression of small cell lung cancer. However, a recent retrospective study of 214 patients in Wuhan reported neurological symptoms in 36.4% of COVID‐19 patients, and the rate rose to 45.5% when the most severely affected patients were considered.^20^ Autopsy of patients who died with COVID‐19 also showed that the brain tissue was hyperemic and edematous, with degeneration of neurons.^21^ Therefore, there is a possibility that the invasion of the nervous system by SARS‐CoV‐2 may be one of the pathogenic mechanisms.^20^, ^22^ Our findings suggests that lung cancer patients may present with neurological manifestation without typical symptoms such as fever and cough at the early stage of COVID‐19. Clinicians should be aware of the possibility of COVID‐19 presenting nonspecifically, including seizures, in order that there will not be a delayed or misdiagnosis.

The main CT findings of lung cancer patients with COVID‐19 have been reported to be similar to radiological findings, commonly reported in patients with COVID‐19.^13^, ^14^, ^23^ We also found some uncommonly reported imaging features. These imaging features may be atypical appearances of general COVID‐19 patients,^23^, ^24^ but they may represent unique appearances of patients with lung cancer.

More importantly, we identified that prolongation of PT on admission was associated with an over two‐fold increased risk of in‐hospital mortality in lung cancer patients with COVID‐19. This finding is in agreement with a previous study demonstrating that coagulation abnormalities may be associated with an increased risk of death in patients with COVID‐19.^25^ The most typical findings reported in patients with COVID‐19 and coagulopathy are an increased D‐dimer concentration, a decrease in platelet count, and a prolongation of PT.^25^, ^26^, ^27^ Although subclinical changes in the coagulation‐fibrinolytic system are often present in lung cancer,^28^, ^29^ coagulation parameters have not previously been studied in lung cancer patients with COVID‐19. In the present study, the nonsurvivors had a prolonged PT, increased D‐dimer concentration, and decreased platelet count, compared to survivors. Based on the findings of the present study and the data from published literature, we recommend closely monitoring coagulopathy in lung patients with COVID‐19 by measuring PT, platelet count, and D‐dimer concentrations.

Several studies have reported that cardiac injury is a common condition among hospitalized patients with COVID‐19, and is associated with a higher risk of in‐hospital mortality^11^, ^30^ The present study also demonstrates that an elevated hs‐TNI on admission was associated with an over seven‐fold increased risk of death in lung cancer patients with COVID‐19. The evidence that SARS‐CoV‐2 directly impairs cardiac function has been demonstrated in a study by Crackower *et al*. in which the targeted disruption of ACE2 in mice resulted in a severe heart contractility defect, increased angiotensin II levels, and upregulation of hypoxia‐induced genes in the heart.^31^

This study has several limitations. First, the number of patients involved in this observational study is relatively small, and thus one should take this limitation into account when interpreting the results and conclusions. Second, from this dataset, we found that lung resection surgery, chemotherapy, and treatment with immune checkpoint inhibitors given within four weeks before the diagnosis of laboratory‐confirmed COVID‐19 were not associated with increased risk with in‐hospital mortality. Similar observations were reported for chemotherapy, immunotherapy, hormonal therapy, targeted therapy, and radiotherapy in cancer patients with COVID‐19 in a study by Lee *et al*.^3^ but the number of lung cancer patients who received active anticancer therapy in their study was small. Further investigation with a greater number of patients will allow us to confirm or refute these findings. Since there has been no previously published study on a large series of lung cancer patients with COVID‐19, and data on clinical, laboratory, and radiographic characteristics lung cancer patients with COVID‐19 are scarce, we believe that this preliminary study is of value in guiding the management of lung cancer patients with COVID‐19.

In conclusion, lung cancer patients with COVID‐19 have high in‐hospital mortality. Prolonged PT and elevated hs‐TNI are independent risk factors for in‐hospital mortality of lung cancer patients with COVID‐19. They may be used to assist clinicians predict prognosis at an early stage for lung cancer patients with COVID‐19.

## Disclosure

All authors have no conflict of interest to declare.

## References

[1] Coronavirus disease (COVID‐19) Situation Report – 162. [cited 30 June 2020.] Available from URL: https://www.who.int/docs/default-source/coronaviruse/20200630-covid-19-sitrep-162.pdf?sfvrsn=e00a5466_2

[2] Kuderer NM , Choueiri TK , Shah DP *et al* Clinical impact of COVID‐19 on patients with cancer (CCC19): A cohort study. Lancet 2020; 6736: 1–13. 10.1016/S0140-6736(20)31187-9 PMC7255743 32473681

[3] Lee LYW , Cazier JB , Starkey T *et al* COVID‐19 mortality in patients with cancer on chemotherapy or other anticancer treatments: A prospective cohort study. Lancet 2020; 395: 1919–26. 32473682 10.1016/S0140-6736(20)31173-9 PMC7255715

[4] Russano M , Citarella F , Vincenzi B , Tonini G , Santini D . Coronavirus disease 2019 or lung cancer: What should we treat? J Thorac Oncol 2020; 15: e105–6. 32283315 10.1016/j.jtho.2020.04.001 PMC7151236

[5] Garassino MC , Whisenant JG , Huang L‐C *et al* COVID‐19 in patients with thoracic malignancies (TERAVOLT): First results of an international, registry‐based, cohort study. Lancet Oncol 2020; 21: 914–22. 32539942 10.1016/S1470-2045(20)30314-4 PMC7292610

[6] Rogado J , Pangua C , Serrano‐montero G *et al* Covid‐19 and lung cancer: A greater fatality rate? Lung Cancer 2020; 146: 19–22. 32505076 10.1016/j.lungcan.2020.05.034 PMC7260554

[7] Zhou F , Yu T , Du R *et al* Clinical course and risk factors for mortality of adult inpatients with COVID‐19 in Wuhan, China: A retrospective cohort study. Lancet 2020; 395: 1054–62. 32171076 10.1016/S0140-6736(20)30566-3 PMC7270627

[8] He W , Chen L , Chen L *et al* COVID‐19 in persons with haematological cancers. Leukemia 2020; 34: 1637–45. 32332856 10.1038/s41375-020-0836-7 PMC7180672

[9] World Health Organization . Clinical management of COVID‐19: interim guidance. [Cited 27 May 2020.] Available from URL: https://www.who.int/publications/i/item/clinical-management-of-covid-19

[10] Singer M , Deutschman CS , Seymour C *et al* The third international consensus definitions for sepsis and septic shock (sepsis‐3). JAMA 2016; 315: 801–10. 26903338 10.1001/jama.2016.0287 PMC4968574

[11] Guo T , Fan Y , Chen M *et al* Cardiovascular implications of fatal outcomes of patients with coronavirus disease 2019 (COVID‐19). JAMA Cardiol 2020; 5: 1–8. 10.1001/jamacardio.2020.1017 PMC7101506 32219356

[12] Huang C , Wang Y , Li X *et al* Clinical features of patients infected with 2019 novel coronavirus in Wuhan, China. Lancet 2020; 395: 497–506. 31986264 10.1016/S0140-6736(20)30183-5 PMC7159299

[13] Song F , Shi N , Shan F *et al* Emerging 2019 novel coronavirus (2019‐NCoV) pneumonia. Radiology 2020; 295: 210–7. 32027573 10.1148/radiol.2020200274 PMC7233366

[14] Zhou Z , Guo D , Li C *et al* Coronavirus disease 2019: Initial chest CT findings. Eur Radiol 2020; 30: 4398–406. 32211963 10.1007/s00330-020-06816-7 PMC7095437

[15] Chen N , Zhou M , Dong X *et al* Epidemiological and clinical characteristics of 99 cases of 2019 novel coronavirus pneumonia in Wuhan, China: A descriptive study. Lancet 2020; 395: 507–13. 32007143 10.1016/S0140-6736(20)30211-7 PMC7135076

[16] Wang D , Hu B , Hu C *et al* Clinical characteristics of 138 hospitalized patients with 2019 novel coronavirus‐infected pneumonia in Wuhan, China. JAMA 2020; 323: 1061–9. 32031570 10.1001/jama.2020.1585 PMC7042881

[17] Onder G , Rezza G , Brusaferro S . Case‐fatality rate and characteristics of patients dying in relation to COVID‐19 in Italy. JAMA 2020; 323: 1775–6. 10.1001/jama.2020.4683. 32203977

[18] Wu Z , McGoogan JM . Characteristics of and important lessons from the coronavirus disease 2019 (COVID‐19) outbreak in China: Summary of a report of 72314 cases from the Chinese Center for Disease Control and Prevention. JAMA 2020; 323: 1239 10.1001/jama.2020.2648. 32091533

[19] Dziewas R , Warnecke T , Zürcher P *et al* Dysphagia in COVID‐19‐multilevel damage to the swallowing network? Eur J Neurol 2020 10.1111/ene.14367. PMC7283711 32460415

[20] Mao L , Jin H , Wang M *et al* Neurologic manifestations of hospitalized patients with coronavirus disease 2019 in Wuhan, China. JAMA Neurol 2020; 77: 1–9. 10.1001/jamaneurol.2020.1127 PMC7149362 32275288

[21] National Health Commission of the People's Republic of China. Diagnosis and treatment of the novel coronavirus pneumonia (Trial version 7) [D]. Published 2020. [3 March 2020.] Available from URL: http://www.nhc.gov.cn/yzygj/s7653p/202003/46c9294a7dfe4cef80dc7f5912eb1989/files/ce3e6945832a438eaae415350a8ce964.pdf

[22] Tassorelli C , Mojoli F , Baldanti F *et al* COVID‐19: What if the brain had a role in causing the deaths? Eur J Neurol 2020; 1–2. 10.1111/ene.14275. PMC7267268 32333819

[23] Simpson S , Kay FU , Abbara S *et al* Radiological Society of North America expert consensus statement on reporting chest CT findings related to COVID‐19. Endorsed by the Society of Thoracic Radiology, the American College of Radiology, and RSNA. J Thorac Imaging 2020; 35: 219–27. 32324653 10.1097/RTI.0000000000000524 PMC7255403

[24] Suppli MH , Riisgaard de Blanck S , Elgaard T , Josipovic M , Pøhl M . Early appearance of COVID‐19 associated pulmonary infiltrates during daily radiotherapy imaging for lung cancer. J Thorac Oncol 2020; 15: 1081–4. 32283316 10.1016/j.jtho.2020.04.004 PMC7151422

[25] Levi M , Thachil J , Iba T , Levy JH . Coagulation abnormalities and thrombosis in patients with COVID‐19. Lancet Haematol 2020; 7: e438–40. 32407672 10.1016/S2352-3026(20)30145-9 PMC7213964

[26] Thachil J , Tang N , Gando S *et al* ISTH interim guidance on recognition and management of coagulopathy in COVID‐19. J Thromb Haemost 2020; 18: 1023–6. 32338827 10.1111/jth.14810 PMC9906133

[27] Tang N , Li D , Wang X , Sun Z . Abnormal coagulation parameters are associated with poor prognosis in patients with novel coronavirus pneumonia. J Thromb Haemost 2020; 18: 844–7. 32073213 10.1111/jth.14768 PMC7166509

[28] Tas F , Kilic L , Serilmez M , Keskin S , Sen F , Duranyildiz D . Clinical and prognostic significance of coagulation assays in lung cancer. Respir Med 2013; 107: 451–7. 23200643 10.1016/j.rmed.2012.11.007

[29] Ferrigno D , Buccheri G , Ricca I . Prognostic significance of blood coagulation tests in lung cancer. Eur Respir J 2001; 17: 667–73. 11401062 10.1183/09031936.01.17406670

[30] Shi S , Qin M , Shen B *et al* Association of cardiac injury with mortality in hospitalized patients with COVID‐19 in Wuhan, China. JAMA Cardiol 2020; 395: 497–506. 10.1001/jamacardio.2020.0950 PMC7097841 32211816

[31] Crackower MA , Sarao R , Oudit GY *et al* Angiotensin‐converting enzyme 2 is an essential regulator of heart function. Nature 2002; 417: 822–8. 12075344 10.1038/nature00786

